# Deficiency of IKK*α* in Macrophages Mitigates Fibrosis Progression in the Kidney after Renal Ischemia-Reperfusion Injury

**DOI:** 10.1155/2021/5521051

**Published:** 2021-12-07

**Authors:** Feng Zhang, Li Fan, Hao Zhang, Wen-juan Huang, Dong Sun, Bin-bin Pan, Xin Wan, Chang-Chun Cao

**Affiliations:** ^1^Department of Nephrology, Nanjing First Hospital, Nanjing Medical University, Nanjing 210006, China; ^2^Department of Nephrology, Sir Run Run Hospital, Nanjing Medical University, Nanjing 211166, China

## Abstract

*Aims*. Acute kidney injury (AKI) can lead to chronic kidney disease (CKD), and macrophages play a key role in this process. The aim of this study was to discover the role of I*κ*B kinase *α* (IKK*α*) in macrophages in the process of AKI-to-CKD transition. *Main Methods*. We crossed lyz2-Cre mice with IKK*α*-floxed mice to generate mice with IKK*α* ablation in macrophages (Mac IKK*α*-/-). A mouse renal ischemia/reperfusion injury (IRI) model was induced by clamping the renal artery for 45 minutes. Treated mice were evaluated for blood biochemistry, tissue histopathology, and fibrosis markers. Macrophages were isolated from the peritoneal cavity for coculturing with tubular epithelial cells (TECs) and flow cytometry analysis. *Key Findings*. We found that fibrosis and kidney function loss after IRI were significantly alleviated in Mac IKK*α*-/- mice compared with wild-type (WT) mice. The expression of fibrosis markers and the infiltration of M2 macrophages were decreased in the kidneys of Mac IKK*α*-/- mice after IRI. The in vitro experiment showed that the IRI TECs cocultured with IKK*α*-/- macrophages (KO M*Φ*s) downregulated the fibrosis markers accompanied by a downregulation of Wnt/*β*-catenin signaling. *Significance*. These data support the hypothesis that IKK*α* is involved in mediating macrophage polarization and increasing the expression of fibrosis-promoting inflammatory factors in macrophages. Therefore, knockdown of IKK*α* in macrophages may be a potential method that can be used to alleviate the AKI-to-CKD transition after IRI.

## 1. Introduction

Chronic kidney disease (CKD) usually leads to end-stage renal disease (ESRD), which is a severe health problem worldwide [1, 2]. CKD is characterized as a progressive loss of renal function with tubular atrophy and tubulointerstitial fibrosis, and the extent of fibrosis is correlated with future functional decline [3]. Because renal tubular epithelial cells (TECs) have the potential for self-repair, it was thought that the function and structure can recover completely after acute kidney injury (AKI). However, patients with AKI who ultimately develop CKD are common, and recent epidemiological studies have demonstrated that AKI contributes to the development of CKD [4]. A meta-analysis showed that patients with AKI had higher risks of developing CKD; the pooled incidence of CKD was 25.8/100 person-years [5].

The mechanism underlying the AKI-to-CKD transition has been studied in-depth in recent years, and the immune response to the infiltration of inflammatory cells was thought to play a key role in the process leading to the AKI-to-CKD transition [6]. Mononuclear macrophage recruitment followed the early infiltration of neutrophils after AKI and lasted for the whole period of recovery. Our previous study and other published studies in which macrophages were depleted before AKI has shown a protective effect against kidney injury [7, 8]. Because of dynamic changes in macrophage phenotypes [9], it is insufficient to study macrophages without distinguishing among the various types. Currently, macrophages are mainly divided into the proinflammatory classically activated (M1) subtype and wound healing/profibrotic alternatively activated (M2) subtype [10, 11]. Furthermore, studies showed that M2 macrophages accumulate in the injured kidney, leading to secretion of profibrotic growth factors, and promote the production of extracellular matrix [9, 12, 13].

Nuclear transcriptional factor NF-*κ*B plays an important role in inflammation [14], and the activation of NF-*κ*B is mediated by I*κ*B kinase (IKK), which consists of two subunits, IKK*α* and IKK*β* [15, 16]. IKK*β* is thought to be an activator of the NF-*κ*B canonical pathway and to induce the production of proinflammatory cytokines, aggravating injury, while IKK*α* provides negative feedback to NF-*κ*B signaling to limit inflammatory gene expression in macrophages [15, 17]. Our previous study and those of others have demonstrated that inhibition of NF-*κ*B or silencing of IKK*β* in an AKI animal model markedly decreased tubule lesions and monocyte/macrophage infiltration [18, 19].

We hypothesized that the activation of IKK*α* could drive the macrophage polarization into the M2 subtype and promote the AKI-to-CKD transition after AKI. In this paper, we established an AKI mouse model with a renal ischemia/reperfusion injury (IRI), and AKI finally led to renal fibrosis. Our study demonstrated that ablation of macrophage IKK*α* ameliorates kidney fibrosis, accompanied by less M2 macrophage infiltration in the kidneys after IRI inflammation, and inhibits the profibrotic pathway in renal TECs.

## 2. Materials and Methods

### 2.1. Animals

Homozygous IKK*α*-floxed mice (C57BL/6 background) were originally obtained from The Jackson Laboratory. Transgenic mice with Cre recombinase controlled by a macrophage-specific Lyz2 promoter (Lyz2-Cre) were obtained from Nanjing Medical University Experimental Animal Center. By mating IKK*α*-floxed mice with Lyz2-Cre transgenic mice, conditional knockout mice with the IKK*α* gene specifically ablated in macrophages (Mac IKK*α*−/− mice) were created. Sex- and age-matched C57BL/6 wild-type (WT) mice (aged 8-12 weeks old and weighing 20-25 g) were bred as controls. All mice were bred under sterile conditions in accordance with guidelines set by the Institutional Animal Care and Use Committee of the Nanjing Medical University. Genotyping was performed by PCR and agarose gel electrophoresis using DNA extracted from mouse tails. The primers used for genotyping were as follows: Cre transgene (sense: 5′-CCCAGAAATGCC AGATTACG-3′; antisense: 5′-CTTGGGCTGCCAGAATTTCTC-3′) and IKK*α*-floxed (sense 1: 5′-CGCTTAGTGTGACTGAGGAAC-3′; sense 2: 5′-ATGAGCCCAACATTTAATCTT-3′; antisense: 5′-GGCATCAGAGTCCGTGGGT-3′).

### 2.2. Renal Ischemia Mouse Model

All research protocols were approved by the Institutional Animal Care and Use Committee of the Nanjing Medical University. Both WT mice and Mac IKK*α*−/− mice were divided into a sham treated group and IRI group; each sham-treated group consisted of 16 mice, and each IRI group had 24 mice. The mice were anesthetized with pentobarbital sodium (40 mg/kg i.p.) and maintained at a temperature of 37°C. After dorsal incision, the left renal pedicle was clamped with a microvascular clamp for 45 minutes, while the sham-operated mice underwent the same treatment except clamping the renal pedicle. The left kidney and blood serum were harvested at day 3, day 7, day 14, and day 21 after IRI; the right kidney was ablated one day before sacrifice; 4 mice from the sham-treated groups and 6 mice from the IRI groups were sacrificed at each time point.

### 2.3. Isolation of Primary Cells and Treatment

We isolated primary TECs according to a previously published method [20] with modifications. The renal tissue from male WT mice (21–30 days) was separated into tubular segments through mechanical grinding and then digested by 0.1% type-2 collagenase for 30 minutes. After digestion, the supernatant was sieved through an 80 *μ*m sieve twice, and the fragments remaining in the sieve were collected and resuspended with 1 : 1 DMEM/F12 culture medium. The cells were seeded onto 24-well plates and cultured at 37°C with 5% CO_2_ in a standard humidified incubator, and the medium was replaced every 2 days.

Isolation of peritoneal macrophages was performed according to a published protocol [21]. Briefly, 1 ml 3% thioglycollate broth (catalog no. 70157; Sigma) was injected into the abdominal cavity of male WT mice or Mac IKK*α*−/− mice (21–30 days). Three days later, the mice were sacrificed and sterilized, 5 ml phosphate-buffered saline (PBS) was injected into the peritoneal cavity of each mouse, the abdomen was massaged for 5 minutes, and then, the peritoneal fluid was harvested. Pooled peritoneal fluid was dispensed into 5 ml tubes and centrifuged at 1000 rpm at 4°C for 5 minutes. The supernatant was discarded, and the cells were resuspended with RPMI 1640 medium. When the primary TECs had grown to 70% confluence, we added CoCl_2_ to the culture medium, and the concentration was adjusted to 100 *μ*mol/l [22]. After treatment for 6 hours to imitate the hypoxia injury, the medium was replaced. Then, the collected primary macrophages, approximately 1 × 10^6^/ml, were placed in a transwell insert (catalog no. MCHT24H48; Merck) on top of primary TECs and cocultured for 72 hours.

### 2.4. Immunofluorescence Staining

Frozen kidney sections of 4 *μ*m thickness were fixed for 20 minutes with 4% paraformaldehyde followed by treatment with 0.2% Triton X-100 to enhance antigen permeability. After blocking in 2% rabbit serum for 60 minutes, sections were incubated overnight at 4°C with Alexa Fluor® 488 anti-mouse CD206 antibody (1 : 100, catalog no. 141709; Biolegend) and PE anti-mouse F4/80 antibody (1 : 100, catalog no. 123109; Biolegend). Ultimately, sections were washed twice with PBS and then viewed with a fluorescence microscopic Olympus IX51; 5 fields of cortical area under ×400 magnification were randomly recorded for each kidney section.

### 2.5. Western Blotting Analysis

The kidneys were lysed with RIPA buffer (Keygen) containing 100 mg/ml PMSF; primary TECs and macrophages were lysed in 1 × SDS sample buffer. The supernatants were collected after centrifugation at 12,000 rpm for 30 minutes, and then, the protein concentration was measured with a BCA protein assay kit (Keygen). A total of 40 *μ*g of lysate proteins was separated on a 10% gel, transferred to a PVDF membrane, blocked with 5% skim milk for 1 hour, and incubated overnight at 4°C with rabbit polyclonal anti-IKK*α* antibody (1 : 10000; catalog no. ab32041; Abcam), anti-*β*-catenin (1 : 1000; catalog no. 9562 s; Cell Signaling Technology), anti-nonphospho (active) *β*-Catenin (1 : 1000; catalog no. 19807; Cell Signaling Technology), anti-Snai1 (1 : 1000; catalog no. 3879 s; Cell Signaling Technology), anti-*α*-SMA (1 : 1000; catalog no. ab5694; Abcam), anti-type I collagen (1 : 2000; catalog no. 14695-1-AP; Proteintech), anti-TNF-*α* (1 : 2000; 17590-1-AP; Proteintech), anti-*β*-actin (catalog no. sc1616; Santa Cruz Biotechnology), anti-IL10 (1 : 1000; catalog no. ab9969; Abcam), anti-Arginase-1 (catalog no. 9819; Cell Signaling Technology), and anti-iNOS Antibody (1 : 2000; catalog no. 18985-1-AP; Proteintech). The next day, after 3 washes in TBST buffer, the membranes were incubated with HRP-conjugated anti-rabbit secondary antibody (1 : 10000, catalog no. sc-2054, Santa Cruz Biotechnology) for 2 hours, and then, the protein bands were detected using an ECL detection system (Bio-Rad, ChemiDoc XRS+). Quantification of the bands was performed by measuring the intensity of the signals by using the ImageJ software package.

### 2.6. Real-Time PCR

Total RNA was extracted from peritoneal macrophages by using RNAiso Plus (catalog no. 9108; Takara) according to the manufacturer's instruction. cDNA was synthesized using 1 *μ*g total RNA and a cDNA Synthesis kit (catalog no. 6110A; Takara) according to the protocol. The expression of mRNA was determined by a Quantitative Real-time Polymerase Chain Reaction (qRT-PCR) kit (Takara, Japan). qRT-PCR was performed by a QuantStudio® 5 Flex Real-Time PCR System (Applied Biosystems, USA) using a SYBR Green kit (Takara, Japan), and the relative changes were quantified using the equation 2 *Δ*CT, in which ΔCT = CTgene–CTcontrol. The following are the primers for iNOS: sense 5′-GTTCTCAGCCCAACAATACAAGA-3′ and antisense 5′-GTGGACGGGTCGATGTCAC-3′. The following are the primers for TNF-*α*: sense 5′-CTGAACTTCGGGGTGATCGG-3′ and antisense 5′-GGCTTGTCACTCGAATTTTGAGA-3′. The following are the primers for IL-10: sense 5′-GCTCTTACTGACTGGCATGAG-3′ and antisense 5′-CGCAGCTCTAGGAGCATGTG-3′. The following are the primers for Arg-1: sense 5′-TTGGGTGGATGCTCACACTG-3′ and antisense 5′-GTACACGATGTCTTTGGCAGA-3′. The following are the primers for *β*-actin: sense 5′-ATATCGCTGCGCTGGTCGTC-3′ and antisense 5′-AGGATGGCGTGAGGGAGAGC-3′.

### 2.7. Immunohistochemical and Immunocytochemical Staining

Formalin-fixed kidney tissues were sliced into 3 *μ*m sections for immunohistochemical staining, and the slides were deparaffinized and rehydrated in a graded alcohol series. The microwave antigen retrieval procedure (citrate buffer, pH 6.0) was performed for 15 minutes. After that, sections were treated with 3% H_2_O_2_ for 20 minutes to eliminate endogenous peroxidase, immersed in 3% rabbit serum for 1 hour to block nonspecific binding sites, and incubated overnight at 4°C with rabbit anti-collagen III antibody (1 : 200, catalog no. 14695-1-AP; Proteintech). The next day, the sections were incubated with horseradish peroxidase- (HRP-) conjugated anti-rabbit secondary antibody for 1 hour at room temperature.

Primary TECs cocultured with control or WT M*Φ*s or KO M*Φ*s were fixed by paraformaldehyde for immunocytochemical staining. The fixed cells were treated with 3% H_2_O_2_ for 20 minutes to eliminate endogenous peroxidase, then immersed in 3% rabbit serum for 1 hour to block nonspecific binding sites, and incubated overnight at 4°C with rabbit anti *α*-SMA antibody (1 : 300; catalog no. ab5694; Abcam). The following treatments were the same as those used for immunohistochemical staining. The positive areas were measured in six randomly selected fields and quantified blindly using ImageJ software.

### 2.8. Flow Cytometry

The peritoneal macrophages isolated from Mac IKK*α*−/− mice or WT mice were washed twice with PBS and centrifuged at 1000 rpm for 5 minutes. Approximately 1 × 10^6^ cells were stained for 30 minutes at room temperature with PE anti-mouse CD14 antibody (123309; Biolegend) and FITC anti-mouse F4/80 antibody (123107; Biolegend) for detecting macrophages, Alexa Fluor® 488 anti-mouse CD206 antibody (141709; Biolegend), and PE anti-mouse F4/80 antibody (123109; Biolegend) for detecting M2 macrophages. Then, the stained cells were washed twice, resuspended in FACS buffer, and analyzed on a Beckman FC 500 Flow Cytometer with FlowJo software.

### 2.9. Histological Examination and Semiquantitative Analyses of the Fibrotic Area in the Kidney Tissue

The renal tissues were fixed in 10% formalin for 24 hours, then embedded in paraffin, and sectioned into 3 mm thick slices. The sections were deparaffinized, rehydrated gradually, and then examined by hematoxylin-eosin (H&E) staining and Masson trichrome staining (catalog no. BA4079B; Baso) according to the manufacturer's protocol. The interstitial area that had undergone fibrosis was stained with aniline blue. We randomly choose nine fields under ×400 magnification from the cortical region of each kidney section. We calculated the average percentage of the fibrotic area in each kidney by measuring the blue area of the selected field with ImageJ software.

### 2.10. Statistical Analysis

Data are expressed as the mean ± s.d. in this study. Comparison between groups was made using analysis of variance (ANOVA). An independent *t*-test was used to compare two groups. The differences were evaluated with SPSS 22.0 software (SPSS). Each experiment was performed at least three times, and two-sided *P* < 0.05 was considered to indicate statistical significance.

## 3. Results

### 3.1. Knockout of IKK*α* in Macrophages Ameliorates the Deterioration of Kidney Function in Mice

**Figure 1:**
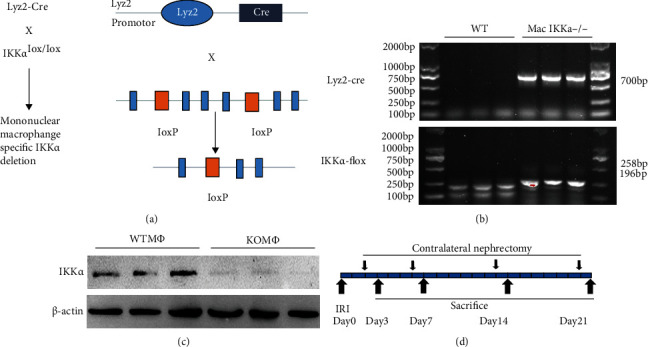
Generation of mice with the IKK*α* gene macrophage-specific ablation and the strategy for the mouse experiment. (a) The strategy of cross-breeding of the IKK*α*-floxed mice (IKK*α* fl/fl) with Cre transgenic mice under the control of a lyz2 promoter (lyz2-Cre). Black boxes indicate the exons of the IKK*α* gene. Orange boxes denote the LoxP sites. (b) PCR analysis for the identification of the genotype of the mice. Lanes 1–3 show the genotyping of the WT mice, while lanes 4–6 indicate the genotypes of Mac IKK*α*−/− mice. (c) Western blotting demonstrated a significantly reduction in the IKK*α* protein of macrophages isolated from Mac IKK*α*−/− mice compared with WT mice after isolation by thioglycollate broth. (d) Strategy for inducing kidney AKI-to-CKD transition: WT and Mac IKK*α*−/− mice were sacrificed at day 3, day 7, day 14, and day 21 after being subjected to renal IR injury.

To investigate the role of macrophage IKK*α* signaling in the AKI-to-CKD transformation, we generated a mouse model with the IKK*α* gene specifically ablated in macrophages (Mac IKK*α*−/−) by utilizing the Cre-LoxP system. Homozygous IKK*α*-floxed mice were mated with lyz2-Cre transgenic mice expressing Cre recombinase under the control of a lyz2 promoter (Figure 1(a)). Conditional knockout mice with Mac IKK*α*−/− were generated, and their genotype was validated by nucleic acid gel electrophoresis (Figure 1(b)). To further confirm the efficacy of the ablation of IKK*α* in macrophages, we isolated macrophages from the peritoneal cavity of the mice and examined IKK*α* expression. As shown in Figure 1(c), Western blot shows a significant reduction in IKK*α* protein in Mac IKK*α*−/− mice compared with the wild-type mice (WT mice). After clamping of the left renal pedicle, we sacrificed mice at day 3, day 7, day 14, and day 21 after the procedure. To detect the function of the injured kidney, the right kidney was ablated one day before sacrifice (Figure 1(d)).

**Figure 2:**
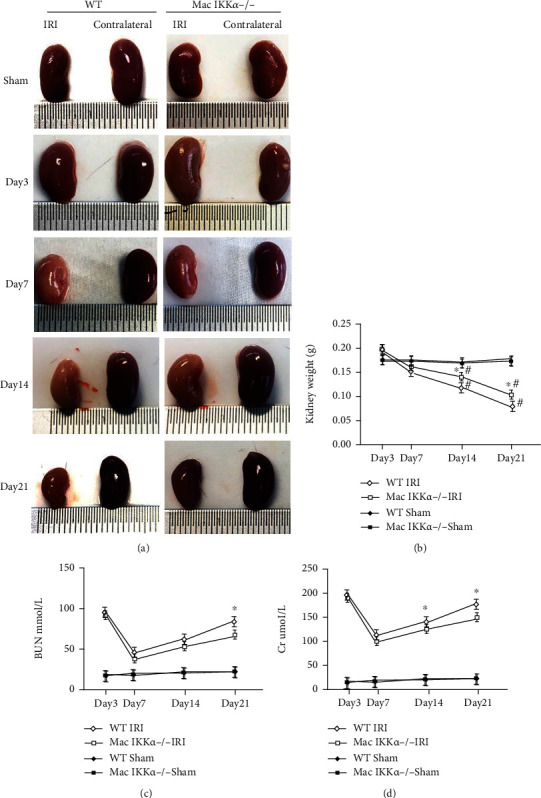
Gross kidney morphology and renal function of WT and Mac IKK*α*−/− mice after IRI. (a) Gross images of kidneys from WT and Mac IKK*α*−/− mice at day 3, day 7, day 14, and day 21 after renal IRI. The IRI kidneys of WT mice were smaller and atrophic compared with kidneys of Mac IKK*α*−/− mice after day 7 following renal IRI. (b) The weight of injured kidneys indicated the extent of atrophy, and the data showed that the kidneys of WT mice were significantly lighter than the kidneys of Mac IKK*α*−/− mice. (c, d) Creatinine and urea nitrogen levels in serum were measured 1 day after contralateral nephrectomy at the indicated time points. Data are presented as the mean ± s.d. ^∗^*P* < 0.05 versus Mac IKK*α*−/− mice; ^#^*P* < 0.05 versus sham-treated mice of the same genotype; *n* = 4-6, two-way ANOVA.

Renal atrophy is associated with the extent of fibrosis, so we recorded the shape of the injured kidneys contrasted by the contralateral untreated kidneys. As shown in Figure 2(a), the untreated kidneys of Mac IKK*α*−/− mice were phenotypically normal, and no obvious abnormality in morphology between WT and Mac IKK*α*−/− mice can be detected. The injured kidneys of both WT and Mac IKK*α*−/− mice were swollen at day 3, and there was no significant difference between them. At day 7 after IRI, the injured kidneys decreased in size and turned gray, which means the kidneys began to undergo fibrosis. Fourteen days after IRI, the kidneys from WT mice were significantly smaller than those from the Mac IKK*α*−/− mice. Because kidney weight is correlated with the extent of atrophy, we measured the weight of the kidneys after sacrificing the mice. As shown in Figure 2(b), the weights of the IRI kidneys of both the WT and KO groups were increased at day 3 and decreased after day 7 compared with those in the sham group, and the WT kidneys became markedly lighter than those of the KO group after day 14. Blood serum creatinine (Cr) and urea nitrogen (BUN) levels were measured to determine kidney function after IRI; the data showed that both Cr and BUN reached a peak at day 3, and there were no significant differences in Cr and BUN levels between the two groups. After day 3, both Cr and BUN levels recovered and reached the low point at day 7, at which point the kidney function of WT mice was markedly worse than that of Mac IKK*α*−/− mice. Interestingly, although kidney function deteriorated again later in the experiment, the levels of Cr and BUN increased without an obvious difference between WT and Mac IKK*α*−/− mice until day 21.

### 3.2. Macrophage-Specific Ablation of IKK*α* Mitigates the Kidney Fibrosis Progression

**Figure 3:**
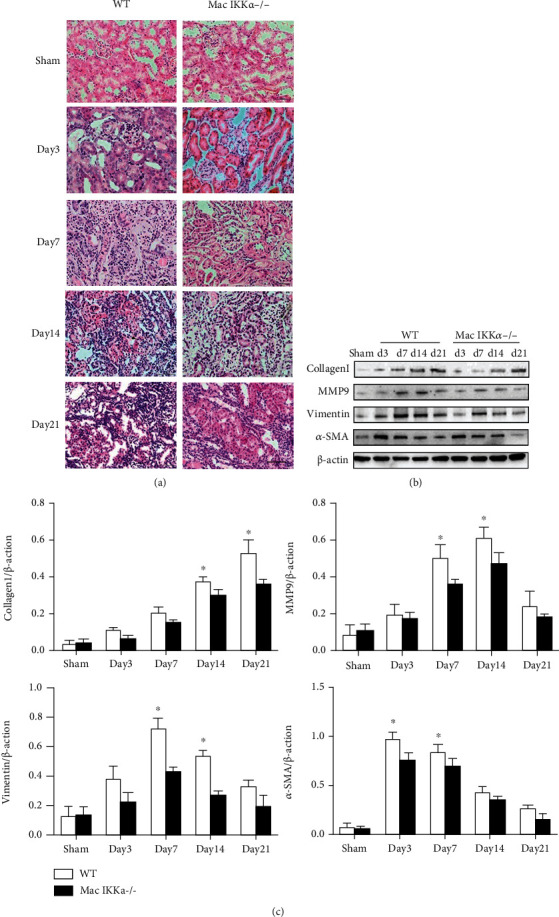
Renal histological changes shown by hematoxylin and eosin staining (H&E) and the expression of proteins that indicate renal fibrosis. (a) Representative images of H&E-stained kidney sections from sham-treated and IRI mice at the indicated time points. Mac IKK*α*−/− mice exhibited a lesser degree of tubular injury than WT mice. Scale bar = 50 *μ*m; magnification, ×400. (b) Representative images of Western blotting for collagen I, MMP9, vimentin, and *α*-SMA in sham kidneys and IRI kidneys at the indicated time points. (c) Expression levels of collagen I, MMP9, vimentin, and *α*-SMA in the kidneys after normalization to *β*-actin as loading controls. Data are presented as the mean ± s.d. ^∗^*P* < 0.05 versus Mac IKK*α*−/− mice; *n* = 4, two-way ANOVA.

**Figure 4:**
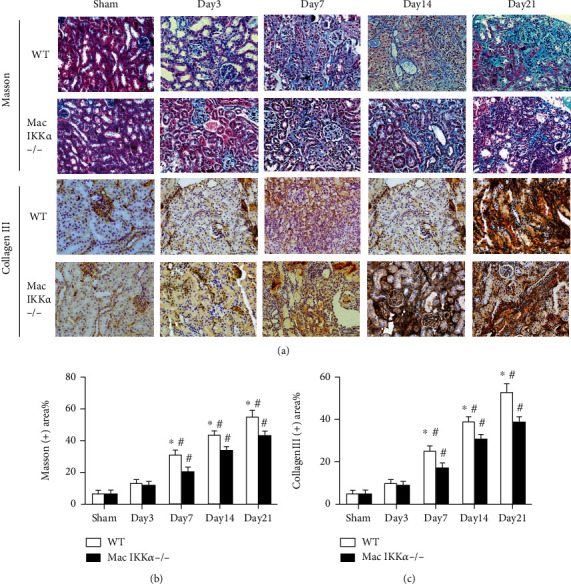
Renal pathological changes in fibrosis. (a, b) Representative images of Masson trichrome staining in kidney sections from sham-treated and IRI mice at the indicated time points. The fibrotic areas increased as time passed, and the extent of fibrosis in WT mice was greater than in Mac IKK*α*−/− mice after day 7 following IRI. Scale bar = 50 *μ*m; magnification, ×400. (a, c) Representative images of immunohistochemical staining of kidney sections for collagen III. The analysis of the images was consistent with Masson trichrome staining. Data are presented as the mean ± s.d. ^∗^*P* < 0.05 versus Mac IKK*α*−/− mice; ^#^*P* < 0.05 versus sham-treated mice of the same genotype; *n* = 4-6, two-way ANOVA.

We then investigated kidney histologic lesions such as tubular atrophy, tubular necrosis, and interstitial extracellular matrix deposition in WT and Mac IKK*α*−/− mice after IRI. As shown in Figure 3(a), there was no obvious difference between the WT and Mac IKK*α*−/− mice in the sham operation group. After day 7 following IRI, the kidneys of the KO Mac IKK*α*−/− mice clearly showed more remnant normal renal tubules than those of the WT mice. We investigated the markers of fibrosis in the kidney after IRI. As shown in Figures 3(b) and 3(c), the accumulation of collagen I had increased by 21 days after IRI, while the level of collagen I was higher in WT mice than in Mac IKK*α*−/− mice, especially after 14 days. Matrix metalloproteinase-9 (MMP9) breaks down the extracellular matrix for the infiltration of inflammatory cells and the proliferation of fibroblasts, and its levels reflect the fibrosis of injured tissue [23, 24]. The data shown in Figure 3(c) indicate that the MMP9 expression was increased at 14 days but decreased at 21 days after IRI. Vimentin and *α*-smooth muscle actin (*α*-SMA) are markers of fibrosis, and their expression typically undergoes an increase followed by a decrease. The level of vimentin and *α*-SMA was higher in the IRI kidneys of WT mice than in Mac IKK*α*−/− mice, meaning that kidneys infiltrated with IKK*α*−/− macrophages underwent moderate fibrotic process after IRI. Immunohistochemical (IHC) staining for collagen III and Masson staining displayed less renal histologic fibrosis in the kidneys of Mac IKK*α*−/− mice than in WT mice at day 7 after IRI (Figures 4(a) and 4(b)).

### 3.3. The Phenotype of the Peritoneal Macrophages Isolated from WT Mice and Mac IKK*α*-/- Mice after Stimulation

**Figure 5:**
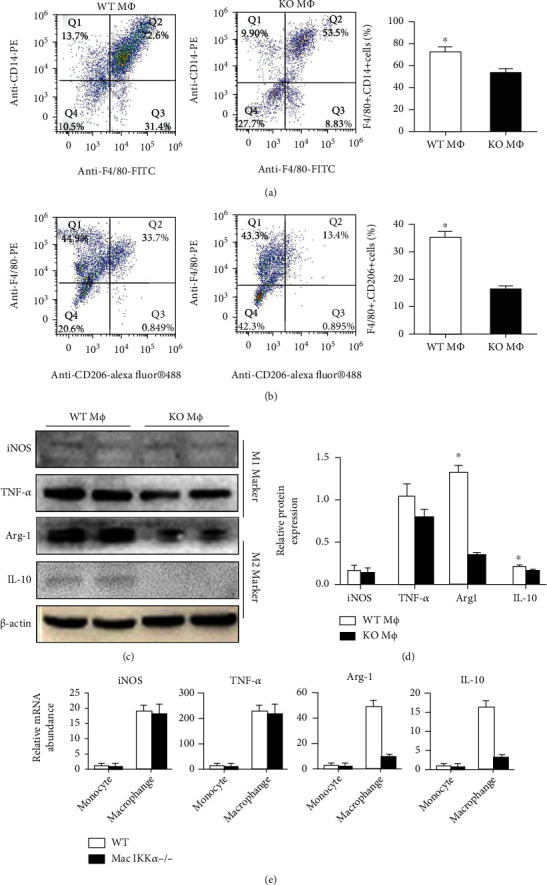
The difference between macrophages of WT mice (WT M*Φ*s) and Mac IKK*α*−/− mice (KO M*Φ*s). Macrophages were induced by injecting thioglycollate broth into the peritoneal cavity of mice for 3 days. (a) The percentage of CD14+ and F4/80+ KO M*Φ*s was smaller than that of WT M*Φ*s assessed by flow cytometry analysis. (b) Representative flow cytometry results showed that the proportion of CD206+ and F4/80+ KO M*Φ*s was smaller than that of WT M*Φ*s. (c) Representative images and quantification of Western blot analyses for expression of iNOS and TNF-*α* as M1 markers and Arg-1 and IL-10 as M2 markers. (d) There was no significant difference in the expression of iNOS and TNF-*α*, while the WT M*Φ*s produced more Arg-1 and IL-10 compared with KO M*Φ*s. Data are presented as the mean ± s.d. ^∗^*P* < 0.05 versus Mac IKK*α*−/− mice; *n* = 3, independent *t*-test. (e) The mRNA abundance for iNOS, TNF-*α*, Arg-1, and IL-10 in monocytes and macrophages isolated from WT mice or Mac IKK*α*−/− mice was assessed; data are presented as the mean ± s.d. ^∗^*P* < 0.05 versus Mac IKK*α*−/− mice; *n* = 3, independent *t*-test.

Inflammatory cells that infiltrate tissues regulate fibrosis after injury, so we suspected that the macrophage polarization after IRI was different in Mac IKK*α*−/− mice than in WT mice. To address this, we examined the macrophage infiltration after induction of intraperitoneal inflammation using flow cytometry (Figures 5(a) and 5(b)). We used CD14 as the marker of mononuclear macrophages, F4/80 as the marker of macrophages, and CD206 as the marker of M2-subtype macrophages [25, 26]. Three days after induction of inflammation, the level of macrophage infiltration in the peritoneal cavity of WT mice was higher than that of Mac IKK*α*−/− mice (Figure 5(a)). To identify the difference between the proportions of macrophages in the two groups, we marked the cells with CD206 and F4/80 simultaneously. The data in Figure 5(b) show that the proportion of CD206+F4/80+ cells in WT mice was larger than in Mac IKK*α*−/− mice (Figure 5(b)). To further investigate the variance in the function of different macrophages, we examined the cytokines secreted by macrophages using Western blotting. The data in Figures 5(c) and 5(d) show that there was no significant difference in iNOS and TNF-*α* as the M1-secreted markers, while the WT M*Φ*s generated more Arg-1 and IL-10, both M2-secreted markers, than KO M*Φ*s.

### 3.4. Infiltration of M2 Macrophages in the Kidney after IRI

**Figure 6:**
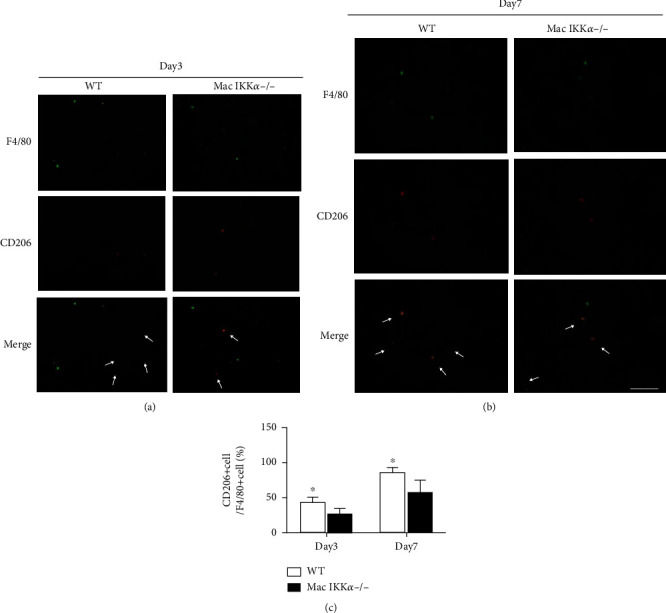
The amount and distribution of M2 macrophages in the kidney after IRI. Mice underwent unilateral renal pedicle clamping for 45 minutes followed by reperfusion. (a) Representative images of dual immunofluorescence staining for F4/80 (green) and CD206 (red) in kidney sections at day 3 after IRI. The proportion of F4/80+CD206+ macrophages in WT kidneys was larger than in Mac IKK*α*−/−kidneys. (b) Dual immunofluorescence staining for F4/80 (green) and CD206 (red) in kidney sections at day 7 after IRI. The cell density of F4/80+CD206+ macrophages in kidney sections was lower compared with the IRI kidney sections at day 3, while more F4/80+CD206+ macrophages can be seen in WT kidneys than Mac IKK*α*−/− kidneys. Scale bars = 50 *μ*m. Magnification, ×400. (c) Quantitative analysis for F4/80+CD206+ macrophages in kidney tissues. White arrows indicate macrophages positive for costaining. ^∗^*P* < 0.05 versus Mac IKK*α*−/− mice; *n* = 5, independent *t*-test.

To explore the differences in macrophage polarization and distribution between WT and Mac IKK*α*−/− mice, sections of injured kidney at day 3 and day 7 were examined by immunofluorescence staining with F4/80 and CD206 as the markers of M2 macrophages. Figures 6(a) and 6(b) show that the subtype of macrophages infiltrating the kidneys changed within 7 days after IRI. The proportion of M2 macrophages increased, while the number of macrophages decreased over time in mice of the same genotype, and the proportion of M2 macrophages in WT mice was higher than that in Mac IKK*α*−/− mice. The number of macrophages at day 14 and day 21 is obviously decreased than that at the first 7 days, while the proportion of M2 macrophages continued to increase. But there was no significant difference between WT and Mac IKK*α*−/− mice after 14 days (Supplemental Figure 1).

### 3.5. Fibrosis Markers and Wnt/*β*-Catenin Pathway in Renal TECs after Coculturing with Macrophages

**Figure 7:**
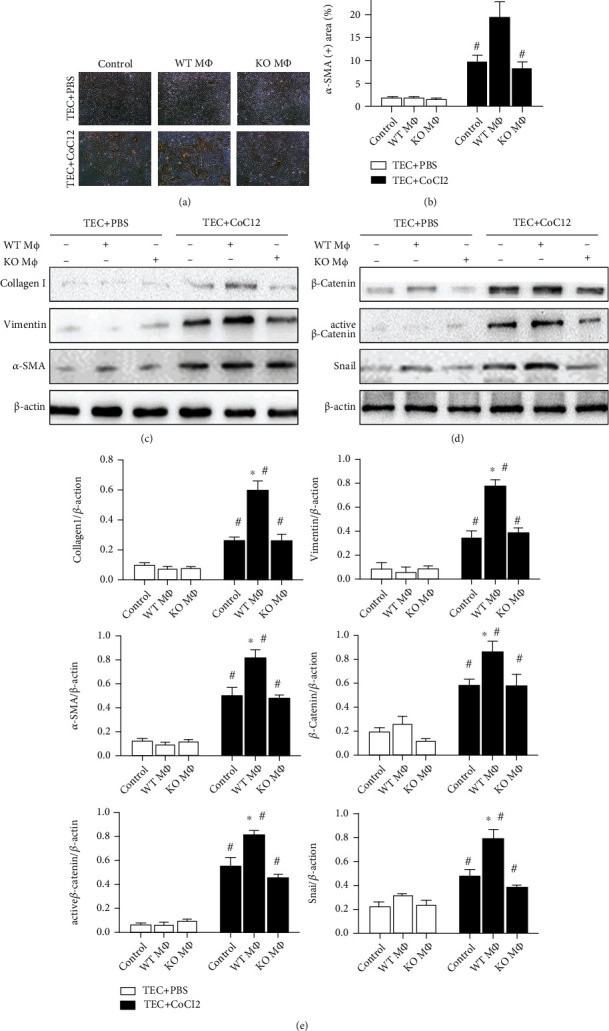
Primary TECs treated with CoCl_2_ to imitate IRI in vitro and then cocultured with WT M*Φ*s or KO M*Φ*s. Then, the fibrosis markers and Wnt/*β*-catenin pathway were investigated. (a) Expression of *α*-SMA by immunohistochemistry in control TECs (top) and IRI TECs (bottom) cocultured with PBS (left), WT M*Φ*s (middle), and KO M*Φ*s (right). Representative images of *α*-SMA staining show distribution in the cytoplasm of TECs that underwent fibrosis. Magnification, ×100. (b) Immunohistochemistry images were measured by ImageJ, and data are presented as the mean ± s.d. ^∗^*P* < 0.05 versus control and coculture with KO M*Φ*s; ^#^*P* < 0.05 versus TECs without IRI; *n* = 3. (c) Protein levels of *α*-SMA, vimentin, and collagen I as fibrosis markers were measured by Western blotting. (d) Protein levels of *β*-catenin, active *β*-catenin, and Snai1 as signals of the Wnt/*β*-catenin pathway were measured by Western blotting. (e) Quantification of Western blot analyses for the proteins mentioned above; the data showed that all fibrosis markers were increased significantly accompanied by Wnt/*β*-catenin pathway activation in IRI TECs cocultured with KO M*Φ*s compared with the WT M*Φ* coculture group and control group, while the expression of the proteins showed no significant difference in TECs without IRI. Data are presented as the mean ± s.d. ^∗^*P* < 0.05 versus control or coculture with KO M*Φ*s; ^#^*P* < 0.05 versus TECs without IRI; *n* = 3, one-way ANOVA followed by the Student-Newman-Keuls test.

To imitate IRI in vitro, we used medium containing CoCl_2_ to culture the renal TECs isolated from WT mice for 6 hours and finally obtained IRI TECs. Then, we cocultured the intraperitoneal macrophages and TECs or IRI TECs for 3 days to investigate the direct impact of macrophages. The IRI TECs showed significantly less proliferation and more morphologic changes than TECs (Figure 7(a)). The immunohistochemical staining of *α*-SMA-positive cells in IRI TECs was greater than that in normal TECs, while IRI TECs cocultured with WT M*Φ*s showed many more *α*-SMA-positive cells than TECs cocultured with KO M*Φ*s. Interestingly, no significant difference in the number of *α*-SMA-positive cells was observed between IRI TECs and IRI TECs cocultured with KO M*Φ*s (Figures 7(a) and 7(b)). Western blotting showed that the expression levels of *α*-SMA, vimentin, and collagen I in TECs receiving different treatments were the same as the *α*-SMA immunohistochemistry results (Figures 7(c) and 7(e)). Because the Wnt/*β*-catenin pathway is the main signaling pathway in the progression of tissue fibrosis [27], we examined the expression of *β*-catenin, active *β*-catenin, and its downstream target Snai1 in IRI TECs cocultured with M*Φ*s (Figures 7(d) and 7(e)). The data showed that the Wnt/*β*-catenin pathway was activated after treatment with CoCl_2_, while coculture with WT M*Φ*s showed more Snai1 expression than control and coculture with KO M*Φ*s.

## 4. Discussion

The infiltration of inflammatory cells in the IRI kidneys in the early phase is alloantigen-independent inflammation, and macrophages are one of the key inflammatory cell contributors to ischemic kidney injury and repair [28]. In this study, we report that IKK*α* could contribute to macrophage M2 polarization. Ablation of IKK*α* in macrophages decreased M2 polarization in macrophages and kidney fibrosis after IRI. Our findings reveal a new mechanism for IKK*α* to mediate the polarization of macrophages in promoting kidney fibrosis in CKD.

NF-*κ*B is a family of dimeric transcription factors that play a crucial role in the inflammatory response and is mediated by the IKK complex, which is composed of two catalytic subunits: IKK*α* and IKK*β* [15, 29]. It is generally accepted that inappropriate activation of the NF-*κ*B pathway is tightly associated with autoimmunity and chronic inflammation. Studies demonstrated that IKK*α* and IKK*β* have distinct functions: IKK*β* plays a key role in canonical NF-*κ*B signaling while IKK*α* is a crucial regulator of noncanonical NF-*κ*B signaling [16, 30]. Accumulated evidence has shown that inhibition of NF-*κ*B signaling by different ways can ameliorate IRI in different organs [31–33]. Previous studies by Cao and colleagues demonstrated that inhibition of IKK*β* activation can significantly attenuate renal IRI in rodent models [19, 34, 35], while IKK*α* in the kidneys promotes the production of anti-inflammatory factors as a negative regulator of canonical NF-*κ*B signaling in the kidneys [36]. It is universally accepted that the resolution of inflammation contributes to kidney recovery following IRI. After IRI, a sterile inflammation induces macrophages to infiltrate and phagocytize cellular debris and produce different cytokines [26]. However, the underlying mechanisms of IKK*α* in macrophages after IRI and the role in leading the AKI-to-CKD transformation remain to be determined. The Lyz2 gene is expressed in myeloid cells in mice; thus, it has been used as a cell-specific marker for myeloblasts, macrophages, and neutrophils [37]. Neutrophil accumulation occurs as early as 30 minutes after IRI, and the role of neutrophils in the pathogenesis of AKI has been controversial [38]. Because neutrophils decreased after 48 h while macrophages infiltration lasted much longer, we choose the 72 h after IRI to minimize the influences of neutrophils.

In this study, a progressive kidney fibrosis model induced by IRI was established by clamping the renal pedicle for 45 minutes. The changes of BUN and Cr demonstrated that the function of the injured kidneys deteriorated to the extreme on the third day and then began to recover until the seventh day. Then, the renal function deteriorated again due to maladaptive repair that means kidney fibrosis gets worse. The mice with IKK*α*-deficient macrophages exhibited better renal function and less renal morphologic atrophy compared with WT mice. Histopathologic examination demonstrated that Mac IKK*α*−/− mice exhibit more normal TECs and less interstitial matrix deposition. This study examined the fibrosis process of TECs both in vivo and in vitro. The in vivo results showed that the different fibrosis proteins were not completely concordant with others as time lapsed, while the kidneys of Mac IKK*α*−/− mice showed reduced expression of defined fibrosis markers such as *α*-SMA compared with WT mice at the same time point.

Because the subphenotype of macrophages is strongly associated with macrophage function [26, 39, 40] and CKD, we investigated the polarization of macrophages after inflammatory stimulation. In the early stage of recovery (day 1 to day 3) after IRI, the deficiency of IKK*α* in macrophages did not lead to differential results; we believed IKK*α* does not play a key role in this stage. In the later stage, macrophages undergo phenotype and functions change in favor of repairing the injured tissue. IKK*α* deficiency in macrophage significantly decreases the M2 macrophage numbers at the site of injury that suggests IKK*α* is required for the conversion of M1 macrophages into M2 which is beneficiary to the fibrosis progress. In our study, the ablation of IKK*α* in macrophages reduced the proportion of M2 macrophages in the peritoneal cavity and inhibited M2 polarization in the kidneys after IRI. Levels of secreted cytokines characteristic of M2 macrophages, such as IL-10 and Arg-1, were significantly downregulated in Mac IKK*α*−/− mice, while expression of proinflammatory factors of M1 macrophages showed no difference. We isolated macrophages from the peritoneal cavity of mice and then cocultured them with primary TECs to exclude the influences from the microenvironment. The results of coculture in vitro showed that macrophages enhanced the expression of fibrosis proteins in TECs that undergo IRI, while the macrophages with ablation of IKK*α* lost the fibrosis-promoting effect. It was interesting that fibrosis did not take place in uninjured TECs whether they were cocultured with PBS or with two types of macrophages, meaning the injury of TECs is indispensable for macrophages to promote the fibrosis process. In vitro, TECs expressed fibrosis protein may be due to epithelial mesenchymal transition proteins (EMT). EMT is a process by which fully differentiated epithelial cells undergo transition to a fibroblast phenotype, generating a matrix [41]; some clinical studies and animal models suggest that EMT plays a role in the pathogenesis of CKD [42, 43]. But a recent study indicated that EMT only is a minor contributor to renal fibrosis; subpopulations of pericytes and fibroblasts are the sources of scar-forming myofibroblasts during kidney fibrosis [44]. In our study, we suspected TECs undergo EMT as accompanying signs of the fibrosis progress.

Wnt/*β*-catenin signaling is activated in various forms of experimental animal models and patients with CKD, and Wnt/*β*-catenin activation induces fibrosis in the kidneys [27, 45]. We examined the key signals of the Wnt/*β*-catenin pathway in primary TECs cocultured with macrophages. The expression of *β*-catenin, nonphospho *β*-catenin as the activated state of *β*-catenin, and Snai1 protein demonstrated that the ablation of IKK*α* in macrophages inhibited the activation of Wnt/*β*-catenin signaling in the kidney after IRI.

## 5. Conclusion

We concluded that IKK*α*-dependent noncanonical NF-*κ*B pathway activation promotes macrophage M2 polarization, which generates cytokine activation of the Wnt/*β*-catenin pathway and ultimately induces fibrosis in the injured kidney. Targeting IKK*α* in macrophages may provide a new strategy for ameliorating progressive kidney disease induced by AKI in patients.

## Data Availability

The data sets used and/or analyzed during the current study are available from the corresponding author on reasonable request.

## Supplementary Materials

Supplementary Materials The amount and distribution of M2 macrophages in the kidney after IRI. Mice underwent unilateral renal pedicle clamping for 45 minutes followed by reperfusion. (A) Representative images of dual immunofluorescence staining for F4/80 (green) and CD206 (red) in kidney sections at day 14 after IRI. There was no significant difference about the proportion of F4/80+CD206+ macrophages between the WT kidneys and Mac IKK*α*−/−kidneys. (B) Dual immunofluorescence staining for F4/80 (green) and CD206 (red) in kidney sections at day 21 after IRI. Scale bars = 50 *μ*m. Magnification: ×400. (C) Quantitative analysis for F4/80+CD206+ macrophages in kidney tissues. *n* = 5; independent *t*-test.
