# Correction to “Deficiency of IKKα in Macrophages Mitigates Fibrosis Progression in the Kidney after Renal Ischemia‐Reperfusion Injury”

**DOI:** 10.1155/jimr/9873896

**Published:** 2026-08-25

F. Zhang, L. Fan, H. Zhang, et al., “Deficiency of IKKα in Macrophages Mitigates Fibrosis Progression in the Kidney after Renal Ischemia‐Reperfusion Injury,” *Journal of Immunology Research*, 2021, 5521051, https://doi.org/10.1155/2021/5521051.

In the article, there is an error in Figure 4, in which the Collagen III/WT Day 14 image in Figure 4a was erroneously duplicated from the Collagen III/WT Day 3 image in the same figure. The figure was correct throughout the peer review process, but the error was introduced during the editing process, when the figure was modified from vertical to horizontal arrangement.

The correct Figure 4 is shown below:

**Figure 4:**
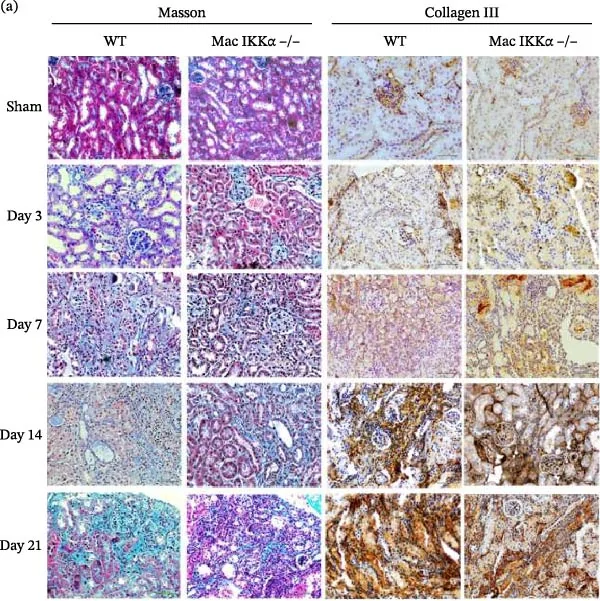
Renal pathological changes in fibrosis. (a, b) Representative images of Masson trichrome staining in kidney sections from sham‐treated and IRI mice at the indicated time points. The fibrotic areas increased as time passed, and the extent of fibrosis in WT mice was greater than in Mac IKKα−/− mice after day 7 following IRI. Scale bar = 50 μm; magnification, ×400. (a, c) Representative images of immunohistochemical staining of kidney sections for collagen III. The analysis of the images was consistent with Masson trichrome staining. Data are presented as the mean ± s:d.  ^∗^ *p* < 0.05 versus Mac IKKα−/− mice; ^#^ *p* < 0.05 versus sham‐treated mice of the same genotype; *n* = 4–6, two‐way ANOVA.

We apologize for this error.

