# Expression of Concern on: “Deficiency of IKK*α* in Macrophages Mitigates Fibrosis Progression in the Kidney after Renal Ischemia‐Reperfusion Injury”

**DOI:** 10.1155/jimr/9803583

**Published:** 2026-09-03

EXPRESSION OF CONCERN: F. Zhang, L. Fan, H. Zhang, et al., “Deficiency of IKK*α* in Macrophages Mitigates Fibrosis Progression in the Kidney after Renal Ischemia‐Reperfusion Injury,” *Journal of Immunology Research*, 2021, 5521051, https://doi.org/10.1155/2021/5521051.

This Expression of Concern is for the above article, published online on 7 December 2021 in Wiley Online Library (wileyonlinelibrary.com) and has been issued by agreement between the Journal Editor‐in‐Chief, Nadja Bakocevic; and John Wiley & Sons Ltd. The Expression of Concern has been agreed due to concerns with the Western blots shown in Figures 3b and 5c, which were identified by the editor during evaluation of the author’s correction request for Figure 4a [1]. Specifically, the rows containing blots for the different investigated proteins in Figures 3b and 5c each have significantly different backgrounds and intensities across lanes. Additionally, no molecular weight markers are presented with which to compare the bands.

The authors reported in their original figure legends that “representative images of Western blotting” were presented, but they could not provide the original Western blots to alleviate the concerns regarding the molecular weights of the proteins analyzed. Therefore, the journal has decided to issue an Expression of Concern to inform and alert readers.
