# Supplementary figures and images for: Deficiency of IKKα in Macrophages Mitigates Fibrosis Progression in the Kidney after Renal Ischemia-Reperfusion Injury
Source: J Immunol Res. 2021 Dec 7;2021:5521051. doi: 10.1155/2021/5521051 (PMC8670970; doi:10.1155/2021/5521051)

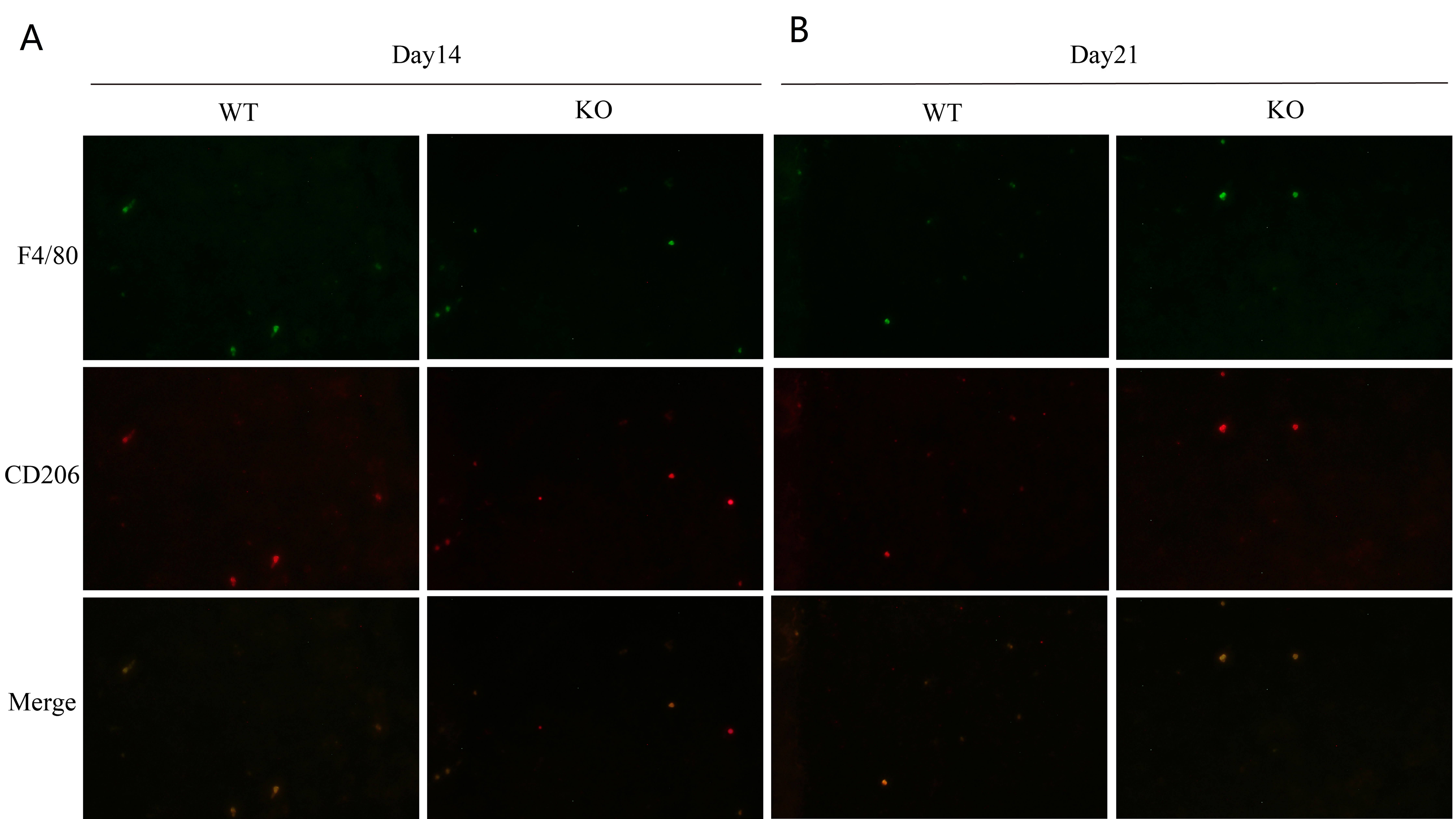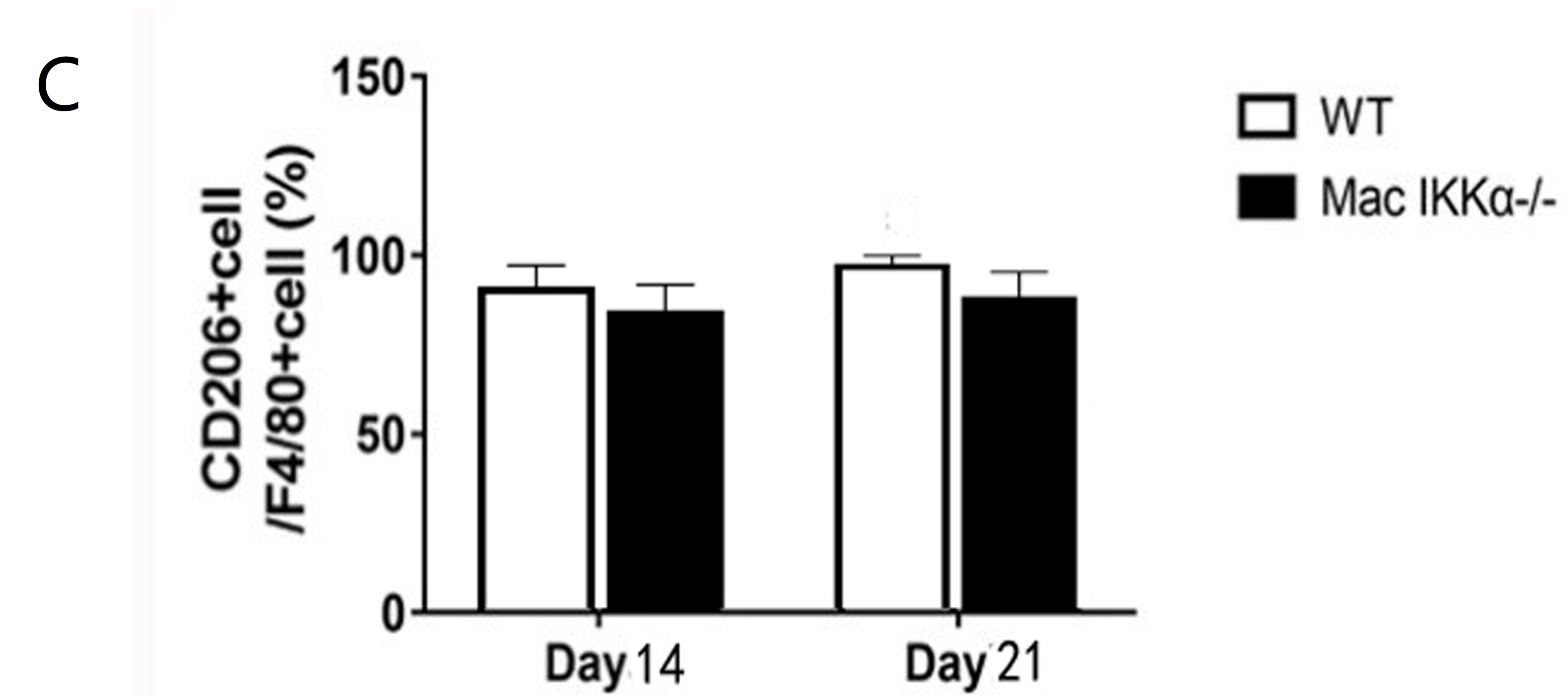

Supplement: Supplementary Materials — The amount and distribution of M2 macrophages in the kidney after IRI. Mice underwent unilateral renal pedicle clamping for 45 minutes followed by reperfusion. (A) Representative images of dual immunofluorescence staining for F4/80 (green) and CD206 (red) in kidney sections at day 14 after IRI. There was no significant difference about the proportion of F4/80+CD206+ macrophages between the WT kidneys and Mac IKKα−/−kidneys. (B) Dual immunofluorescence staining for F4/80 (green) and CD206 (red) in kidney sections at day 21 after IRI. Scale bars = 50 μm. Magnification: ×400. (C) Quantitative analysis for F4/80+CD206+ macrophages in kidney tissues. n = 5; independent t-test. [file 5521051.f1.pdf]
